# Online Purchase Attempts of Flavored E-Cigarettes to Minors in California Before and After Senate Bill 793

**DOI:** 10.1001/jamanetworkopen.2023.48749

**Published:** 2023-12-21

**Authors:** Scott I. Donaldson, Trista A. Beard, Rafael Colonna, Elizabeth Andersen-Rodgers, Heather L. Wipfli, Kurt M. Ribisl, Jon-Patrick Allem

**Affiliations:** 1 Department of Population and Public Health Sciences, Keck School of Medicine, University of Southern California, Los Angeles; 2 California Tobacco Prevention Program, California Department of Public Health, Sacramento; 3 Department of Health Behavior, Gillings School of Global Public Health, University of North Carolina at Chapel Hill; 4 Department of Health Behavior, Society and Policy, Rutgers School of Public Health, The State University of New Jersey, New Brunswick

## Abstract

This survey study assesses whether the online purchase attempt completion rate of e-cigarettes changed after passage of California Senate Bill 793.

## Introduction

E-cigarette use among adolescents has been considered a global public health concern.^1^ To prevent youth access to flavored e-cigarette products, California enacted Senate Bill (SB) 793 on December 21, 2022, prohibiting the sale of most flavored tobacco products, including e-cigarettes, while exempting hookah, premium cigars, and loose-leaf tobacco.^2^ This survey study examined attempted online purchases of flavored e-cigarette products to researchers posing as minors (age <21 years) before and after SB 793.

## Methods

This study included websites of 6 leading e-cigarette brands and 20 e-cigarette vendors in California (eTable 1 in Supplement 1).^3^ The eMethods and eTable 2 in Supplement 1 indicate how vendors were selected. Four adult researchers posing as minors visited all 26 websites from April 4 through May 5, 2022 (before SB 793) and from February 4 through March 26, 2023 (after SB 793). The study was approved by the University of Southern California institutional review board.

A purchase attempt was defined as a researcher’s ability to add a flavored e-cigarette product to their cart and, when necessary, pass the age verification system (the eMethods in Supplement 1 explains how age verification systems were determined) and provide their credit card information. Researchers coded for 3 e-cigarette product types (the eMethods in Supplement 1 details product types). Researchers were instructed to add the first flavored e-cigarette product (the eMethods in Supplement 1 gives flavor categories) that they noticed on the website for each product type to their carts (if available).

To test for statistical significance between data from before vs after SB 793, a series of 2-tailed nonparametric Wilcoxon signed rank tests were conducted. To correct for multiplicity, *P* = .003 (α = .05/15) was used to determine statistical significance. The rate (ie, number of completed attempts/total attempts) of completed purchase attempts of flavored e-cigarette products by product type was computed before and after SB 793. Data were analyzed using SPSS, version 29.0.1.0.

## Results

Before SB 793, the overall flavored e-cigarette purchase attempt success rate was 52.2% (12 of 23 attempts), while after SB 793, the overall rate was 60.9% (14 of 23 attempts) (*P* = .16). The Table shows the rates of completed purchase attempts of flavored e-cigarette products to minors before and after SB 793 by e-cigarette product type.

**Table.** Rates of Completed Online Purchase Attempts of Flavored E-Cigarettes to Researchers Posing as Minors Before and After SB 793

Flavored e-cigarette product	Completed attempts, No./total No. (%)		*P* value
	Before SB 793	After SB 793	
Second-generation e-cigarette	2/4 (50.0)	2/3 (66.7)	>.99
JUUL	1/4 (25.0)	3/5 (60.0)	.16
Disposable e-cigarette	9/15 (60.0)	9/15 (60.0)	>.99
All	12/23 (52.2)	14/23 (60.9)	.16

Abbreviation: SB, Senate Bill.

## Discussion

No statistically significant differences were observed between the pre–SB 793 and post–SB 793 periods in the rate of purchase attempts of flavored e-cigarettes, suggesting that SB 793 has yet to influence online retailers. It remains unclear as to why this is the case. Future research should include a comprehensive evaluation of SB 793 compliance among brands and vendors that sell products online to California consumers. This research could provide data on retailer awareness of the new legislation and perceptions of the consequences of violating the new flavor ban. Vendors may be flouting the new law, may be ignorant of the new law, or may not believe that the new law applies to online sales.

This study was restricted to 20 vendors and 6 brands that sell e-cigarette products online in California, limiting its generalizability. This study’s protocol was developed during the COVID-19 pandemic. With the health and safety of the research team prioritized, this study avoided face-to-face interactions. As a result, orders were not submitted in this study, precluding the research team’s ability to examine whether age was verified at delivery and to calculate the actual purchase rate. However, 4 websites (15.4%) in this study stated that age verification would occur at delivery. These findings warrant urgent attention from decision-makers to enforce the ban on flavored e-cigarette products in California.

## Notes

Supplement 1.
eMethods.
**eTable 1.** List of 6 leading e-cigarette brand and 20 e-cigarette vendor websites in California **eTable 2.** County-level breakdown of e-cigarette vendor Yelp search strategy
eReferences.

Supplement 2.
Data Sharing Statement
