# Supplementary material for: Online Purchase Attempts of Flavored E-Cigarettes to Minors in California Before and After Senate Bill 793
Source: JAMA Netw Open. 2023 Dec 21;6(12):e2348749. doi: 10.1001/jamanetworkopen.2023.48749 (PMC10739096; doi:10.1001/jamanetworkopen.2023.48749)
Supplement: Supplement 1. — eMethods. eTable 1. List of 6 leading e-cigarette brand and 20 e-cigarette vendor websites in California eTable 2. County-level breakdown of e-cigarette vendor Yelp search strategy eReferences. [file jamanetwopen-e2348749-s001.pdf]

Supplemental Online Content

Donaldson SI, Beard TA, Colonna R, et al. Online purchase attempts of flavored e-cigarettes to minors in California before and after Senate Bill 793. *JAMA Netw Open.* 2023;6(12):e2348749. doi:10.1001/jamanetworkopen.2023.48749

eMethods.

eTable 1. List of 6 leading e-cigarette brand and 20 e-cigarette vendor websites in California

eTable 2. County-level breakdown of e-cigarette vendor Yelp search strategy

eReferences.

This supplemental material has been provided by the authors to give readers additional information about their work.

© 2023 Donaldson SI et al. *JAMA Network Open.*

eMethods.
Vendor selection

E-cigarette vendors (i.e., those specialized in selling e-cigarettes but may also carry other products like CBD) from five regions in California (i.e., Southern Coastal, Los Angeles Coastal, Southern Inland, Central, and Northern) were searched and sampled (**eTable 1**). To be included in this study, each e-cigarette vendor had to have a retail store with a physical address in California and a live website. The research team entered the search term "electronic cigarettes" on Yelp (www.yelp.com), filtered by the county or city of interest, and sorted by "recommended." Yelp recommendations consider a user's search terms, distance from the city entered into the Yelp search engine, ratings, reviews, and user engagement data (e.g., transactions made through Yelp).[1] Yelp includes a diverse sample of e-cigarette vendors and has been used to identify e-cigarette retailers.[2,3] To identify official retailers, members of the research team cross-referenced the list provided by Yelp with all licensed California tobacco product retailers registered with the California Department of Tax and Fee Administration (CDTFA).[4] Due to taxpayer confidentiality laws, no personal data were included. The study team selected between 3-7 e-cigarette vendors from each region in California (i.e., Los Angeles Coastal (n=7), Southern Coastal (n=3), Southern Inland (n=3), Central (n=4), Northern (n=3), totaling (n=20) e-cigarette vendors (**eTable 2**).

Brand selection

The study team selected 6 leading e-cigarette brands with live websites based on market share data in 2020,[5] including JUUL, Puff Bar, Blu, NJOY, Vuse, and Innokin.

Product types

Researchers were given definitions for three e-cigarette product types and coded for 1) second-generation e-cigarettes (i.e., e-cigarettes that have a battery, a heating element, and a place to hold e-liquid), 2) JUUL brand products (i.e., products solely branded by JUUL and distinct from older-generation e-cigarette models that tended to be larger, less discrete, and more expensive), and 3) disposable e-cigarettes (i.e., small, portable, pre-filled with e-liquid, and not rechargeable).

Flavors

Coded flavor categories included fruit, sweet, menthol, liquor, or concept flavors (e.g., vague non-characterizing flavors like "Sunset Lava"). Tobacco (e.g., original, bold) was not coded as a flavored product.

Age verification mechanisms

Based on past studies,[6,7] age verification mechanisms were coded at checkout and ranked on a continuum from ineffective to effective. For example, ineffective mechanisms included statements that: 1) clicking a checkbox or button indicates that the customer is over 21, 2) by

© 2023 Donaldson SI et al. *JAMA Network Open.*

submitting the order the customer certifies their age is over 21, 3) birthdate is required, and 4) no age verification mechanism was used. Age verification mechanisms that could be effective include: 5) public records (i.e., personal information verified using third-party software), 6) challenge questions (i.e., multiple-choice questions based on public records like “With which company do you have a mortgage?”), 7) age is verified at delivery, and 8) upload or enter a social security number.

Coding reliability

Five e-cigarette websites were double coded to establish interrater reliability on available e-cigarette product types and age verification mechanisms. The average percent agreement scores ranged from 78.6% to 96.7%, which is considered acceptable.[8] All disagreements were resolved by an arbitrator.

eReferences

1. Yelp Support Center. How does Yelp determine its search results? Published June 23, 2022. Accessed June 23, 2022. https://www.yelp-support.com/article/How-does-Yelp-determine-its-search-results?l=en_US

2. Sussman S, Garcia R, Cruz TB, et al. Consumers’ perceptions of vape shops in Southern California: an analysis of online Yelp reviews. *Tob Induc Dis*. 2014;12(1). doi:10.1186/s12971-014-0022-7

3. Galstyan E, Galimov A, Meza L, et al. An assessment of vape shop products in California before and after implementation of FDA and state regulations. *Int J Environ Res Public Health*. 2022;19(23):15827. doi:10.3390/ijerph192315827

4. California Department of Tax and Fee Administration. California cigarette & tobacco products licensees. Published May 12, 2021. Accessed May 12, 2021. https://www.cdtfa.ca.gov/taxes-and-fees/cigarette-licensees.htm

5. Statista. Dollar share of e-cigarette sales in the United States in 2022. Published 2022. Accessed October 5, 2022. https://www.statista.com/statistics/1097004/e-cigarette-market-share-us-by-brand/

6. Williams RS, Derrick J, Ribisl KM. Electronic cigarette sales to minors via the internet. *JAMA Pediatr*. 2015;169(3):e1563. doi:10.1001/jamapediatrics.2015.63

7. Williams RS, Phillips-Weiner KJ, Vincus AA. Age verification and online sales of little cigars and cigarillos to minors. *Tob Regul Sci*. 2020;6(2):152-163. doi:10.18001/trs.6.2.6

8. Watson PF, Petrie A. Method agreement analysis: a review of correct methodology. *Theriogenology*. 2010;73(9):1167-1179. doi:10.1016/j.theriogenology.2010.01.003

© 2023 Donaldson SI et al. *JAMA Network Open.*

eTable 1. List of 6 leading e-cigarette brand and 20 e-cigarette vendor websites in California	
**Brand**	**Website Link**
JUUL	https://www.juul.com/
Puff Bar	https://puffbar.com/
Blu	https://www.blu.com/en/US
NJOY	https://njoy.com/us/
Vuse	https://vusevapor.com/
Innokin	https://www.innokin.com/
**Vendor**	**Website Link**
Stogz	https://stogz.com/
Yours Truly CBD	https://www.yourstrulycbd.com/
The Vape Shop	https://www.thevapeshopla.com/
V for Vape	https://vforvape.com/
Teagardins	https://www.tgrsupply.com/
Vapor Forrest	https://vaporforrest.store/
Vape Juice Depot	https://vapejuicedepot.com/
Vapor Empire	https://vaporempire.com/
Upscale Vapes	https://upscalevapes.com/
Epik Vapes	http://epikvape.com/
Vape3One	https://www.vape3one.com/
San Jose Vape	https://www.caminovapes.com/
Ziggys Smoke Shop	https://ziggyssmokeshops.com/
Vapor Den	https://vaporden.com/
Millbrae Vape	https://millbraevapes.myshopify.com/
C3 Vapor	https://c3vapors.com/
V Spot Vapor	https://www.vspotvapor.com/
Stay Vaped	https://www.stayvaped.com/
Smokeless Vape and CBD	https://www.smokelessfolsom.com/
Apollo Vapes	https://www.apolloecigs.com/

eTable 2. County-level breakdown of e-cigarette vendor Yelp search strategy	
**Region 1:**	Southern Coastal (Orange, and San Diego counties)
**Region 2:**	Los Angeles Coastal (Los Angeles, San Luis Obispo, Santa Barbara, and Ventura counties)
**Region 3:**	Southern Inland (Imperial, Riverside, San Bernardino, and Kern counties)
**Region 4:**	Central (Monterey, Kings, Merced, Santa Clara, Santa Cruz, San Mateo, Stanislaus, Mariposa, Medera, Fresno, San Benito, and Inyo counties)
**Region 5:**	Northern (Calaveras, El Dorado, Nevada, Plumas, Napa, Contra Costa, Mono, Sacramento, Trinity, Modoc, Placer, San Joaquin, Tuloumne, Butte, Alameda, Sutter, Shasta, Yuba, Glen, Lassen, Del Norte, Solano, Sonoma, Humboldt, Siskiou, Colusa, Marin, Mendocino, Yolo, Lake, Amador, San Francisco, Alpine, Sierra, and Tehama counties)

© 2023 Donaldson SI et al. *JAMA Network Open.*
