# Supplementary material for: Online Purchase Attempts of Flavored E-Cigarettes to Minors in California Before and After Senate Bill 793
Source: JAMA Netw Open. 2023 Dec 21;6(12):e2348749. doi: 10.1001/jamanetworkopen.2023.48749 (PMC10739096; doi:10.1001/jamanetworkopen.2023.48749)
Supplement: Supplement 2. — Data Sharing Statement [file jamanetwopen-e2348749-s002.pdf]

Data Sharing Statement

Donaldson. Online Purchase Attempts of Flavored E-Cigarettes to Minors in California Before and After Senate Bill 793. *JAMA Netw Open*. Published December 21, 2023. doi:10.1001/jamanetworkopen.2023.48749

Data
Data available: Yes
Data types: Data (not involving human participants)
How to access data: jon.patrick.allem@rutgers.edu
When available: With publication

Supporting Documents
Document types: None

Additional Information
Who can access the data: jon.patrick.allem@rutgers.edu
Types of analyses: for any purpose
Mechanisms of data availability: with investigator support
